# Common oscillatory mechanisms across multiple memory systems

**DOI:** 10.1038/s41539-016-0001-2

**Published:** 2017-01-05

**Authors:** Drew B. Headley, Denis Paré

**Affiliations:** Center for Molecular and Behavioral Neuroscience, Rutgers University, Newark, NJ 07102 USA

## Abstract

The cortex, hippocampus, and striatum support dissociable forms of memory. While each of these regions contains specialized circuitry supporting their respective functions, all structure their activities across time with delta, theta, and gamma rhythms. We review how these oscillations are generated and how they coordinate distinct memory systems during encoding, consolidation, and retrieval. First, gamma oscillations occur in all regions and coordinate local spiking, compressing it into short population bursts. Second, gamma oscillations are modulated by delta and theta oscillations. Third, oscillatory dynamics in these memory systems can operate in either a “slow” or “fast” mode. The slow mode happens during slow-wave sleep and is characterized by large irregular activity in the hippocampus and delta oscillations in cortical and striatal circuits. The fast mode occurs during active waking and rapid eye movement (REM) sleep and is characterized by theta oscillations in the hippocampus and its targets, along with gamma oscillations in the rest of cortex. In waking, the fast mode is associated with the efficacious encoding and retrieval of declarative and procedural memories. Theta and gamma oscillations have similar relationships with encoding and retrieval across multiple forms of memory and brain regions, despite regional differences in microcircuitry and information content. Differences in the oscillatory coordination of memory systems during sleep might explain why the consolidation of some forms of memory is sensitive to slow-wave sleep, while others depend on REM. In particular, theta oscillations appear to support the consolidation of certain types of procedural memories during REM, while delta oscillations during slow-wave sleep seem to promote declarative and procedural memories.

## Introduction

Different forms of memory are supported by the hippocampus, striatum, and cortex (here defined to include the neocortex and parahippocampal regions). While each of these regions has specialized anatomical and response properties that may support their respective mnemonic capabilities, they also share similar patterns of oscillatory potentials, which are thought to facilitate local processing and inter-regional communication.^1^ This review will show that these different brain regions and types of memory share common oscillations that are similarly associated with the encoding, consolidation, and retrieval of memories. This review will first cover the multiple forms of memory and brain regions to be discussed. Then the focus will shift to the mechanisms underlying each oscillation. Next we will examine how across different regions and forms of memory, certain oscillations are associated with particular stages of memory processing. Finally, the case will be put forward that, in general, different subsets of rhythms can be linked to either the encoding and retrieval or consolidation of memories.

## Multiple memory systems

Memories can take a variety of forms, but are often divided into two broad categories, procedural and declarative.^2^ Within the declarative type, a further subdivision is made between episodic memories for events an individual experienced in a particular spatiotemporal context, and semantic memories for facts and things that are divorced from any particular episode. Procedural memory encompasses a wide range of phenomena, such as motor and perceptual skills, habits, unconscious changes in response tendencies (e.g., priming), and simple classical conditioning. However, in any given situation, the demarcation between these distinct forms of memory is not always clear. For instance, when subjects are trained to associate a tone with an aversive airpuff to the eye, they acquire both an episodic memory for the circumstances of the training and a conditioned reflex to the tone, with the two depending on dissociable neural substrates.^3^ This specialization may have arisen because different forms of memory place incompatible computational demands on neural systems.^4^ A particular circuit’s performance on one kind of memory may be compromised by adding the functionality that supports other types of memories and their associated behaviors. Yet, at a basic level, these circuits often share similar cell types and synaptic physiology. Those common low-level mechanisms impose constraints on the spatiotemporal integration and transmission of neural activity. Emerging from these constraints are the various oscillations we will cover. They may serve as general-purpose mechanisms that enable the efficacious encoding and propagation of activities within and between networks, while at the same time being blind to their information content.

## Three memory systems: Hippocampus, cortex, and striatum

Starting with pioneering studies on patient HM,^5^ it was recognized that the hippocampus is important for the rich encoding of novel experiences. Similarly in rodents, damage to the hippocampus impairs acquisition of contextual fear memory,^6^ a task that requires the rapid formation of long-term associations between multiple sensory modalities. In addition, the hippocampus’ role in memory is often time-limited.^6^ This has been attributed to a systems’ consolidation process, whereby memories are gradually transferred from the hippocampus to the cortex for long-term storage.^7^ Studies have found that disrupting neocortical activity at specific sites can impair the retrieval, consolidation, and encoding of memories for the specific contents they represent.^8–10^ Correspondingly, cortical regions also exhibit experience-dependent structural and functional plasticity.^11–13^

While the cortex and hippocampus are associated with declarative memory, the striatum is linked to several types of procedural memory.^14^ These include skilled motor behaviors, such as performing a particular sequence of hand movements. Associations between specific stimuli and responses can also depend on the striatum, especially after extensive training that renders such behavior “habitual”. Both of these forms of learning contrast with declarative memories in that they are often implicit (subjects can show these memories without conscious awareness). This learning is also inflexible: motor learning does not readily transfer to novel sequences of actions; habits are rather stimulus-specific.

## Three oscillations: delta, theta, and gamma

**Fig. 1:**
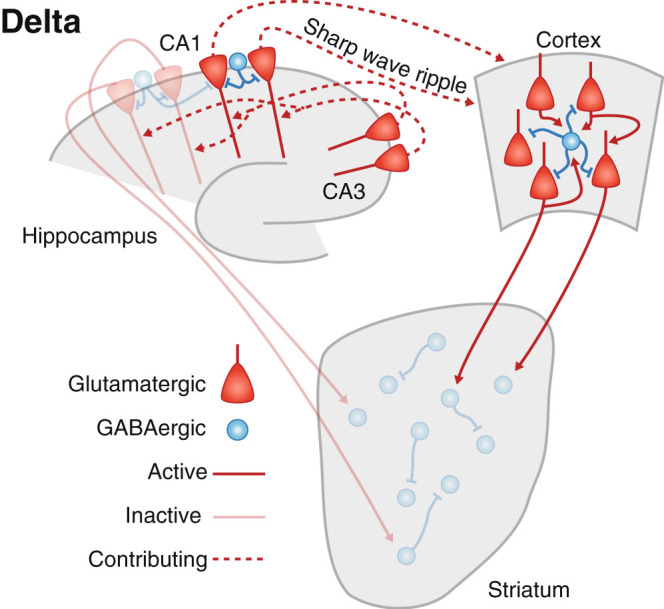
Delta oscillations arise from the propagation of activity throughout the cortex. SWRs in the hippocampus are correlated with the phase of delta oscillations. Striatum also exhibits delta that is driven by cortical inputs

Neuronal oscillations are thought to coordinate activity into ordered patterns that optimize local information processing and facilitate signal transmission between regions.^1^ We will consider three distinct rhythms, delta (0.5–2 Hz), theta (6–9 Hz), and gamma (35–100 Hz), that can be recorded from, or have been shown to influence the cortex, hippocampus, and striatum (Fig. 1). While delta is associated with slow-wave sleep (SWS) and theta is prominent during active waking and rapid eye movement (REM) sleep, both modulate the power of the faster gamma oscillation (reviewed below). Of note, nested within delta are several distinct oscillatory phenomena, such as spindling (7–14 Hz) and faster forms of delta (2–4 Hz) that are not discussed here. Readers familiar with the phenomenology and origin of neuronal oscillations may wish to skip the next three subsections and jump to “Oscillations and memory systems”.

## Generation of delta oscillations

Sleep generally comprises two distinct states, SWS and REM. Delta oscillations are present in SWS and reflect alternating periods of depolarization and hyperpolarization that are synchronized across wide swathes of cortex.^15^ While delta oscillations are regulated by brainstem centers,^16^ and recruit multiple subcortical sites, including the dorsal striatum^17^ and thalamus,^18^ the intrinsic cortical circuitry is sufficient for their generation.^15,19^

The periods of tonic depolarization and hyperpolarization that comprise these slow waves have come to be referred as to up- and downstates, respectively (see ref. 20 for an excellent review). Upstates reflect a barrage of excitatory post-synaptic potentials and inhibitory post-synaptic potentials that cause a substantial increase in membrane conductance,^21^ while downstates are associated with reduced synaptic activity and disfacilitation. During upstates, the mixture of excitatory and inhibitory inputs imposes a “balanced” state that favors the genesis of gamma oscillations.^21,22^

While up- and downstates have received the most attention in cortex, the terms “up-” and “down”-state were first introduced to describe the behavior of striatal medium spiny neurons (MSNs) in vivo.^17,23^ As in cortex, up- and downstates are synchronized between distant striatal sites.^24^ Unlike cortex, striatal upstates are not generated by local circuit interactions within the striatum, but imposed by corticostriatal neurons driving striatal interneurons and MSNs.^23,25,26^

**Fig. 2:**
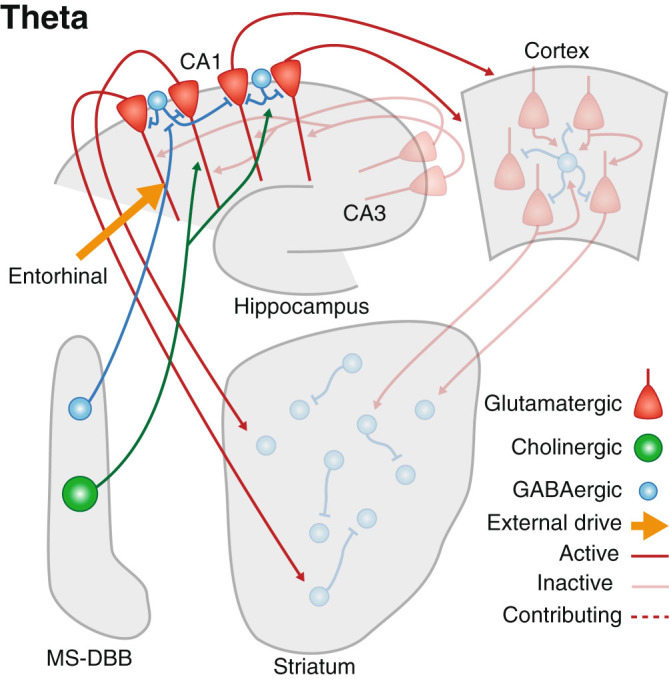
Theta oscillations depend upon pacing from the MS-DBB and excitatory drive from the entorhinal cortex. Both the striatum and cortical regions receiving input from CA1/subiculum that show theta as well

Cortical delta oscillations also interact with hippocampal activity. Dentate granule cells, CA1 pyramidal neurons, and interneurons of the stratum radiatum and stratum lacunosum moleculare, all of which receive direct inputs from entorhinal cortex, exhibit subthreshold modulation of their membrane potential synchronized with cortical delta oscillations.^27–29^ Compared with their cortical and striatal counterparts, these modulations tend to be weaker and lack the clear bimodality found during cortical up- and downstates, although they can entrain spiking.^27,30^ Instead, during SWS the hippocampus mostly expresses large irregular activity,^31^ including sharp wave ripples (SWRs), which result from the synchronous activation of CA1 pyramidal cells and interneurons by CA3 afferents.^32^ SWRs tend to occur at the onset of upstates,^27,33,34^ and are thought to play an important role in memory consolidation by reactivating hippocampal and cortical activity patterns that occurred during past experiences.^35^ Multiple lines of evidence have converged to support their role in memory consolidation.^36^ Moreover, there is evidence that cortical reactivations during upstates may be driven by temporally proximal SWRs.^34^^,^^37^ In addition to their role in offline consolidation, SWRs also occur during pauses in task performance, coordinating the replay of prior trajectories by hippocampal place cells, in both the forward and reverse orders.^38,39^ Disrupting these online SWRs impairs performance on a task requiring spatial working memory (Fig. 2).^39^

## Generation of theta oscillations

Theta oscillations are strongest in the hippocampus during movement, exploration, sniffing, and REM sleep.^31^ For the most part, theta generation depends upon the medial septum-diagonal band of broca (MS-DBB).^40^ The MS-DBB contains cholinergic, GABAergic, and glutamatergic projection neurons. Injection of anticholinergic drugs or toxins selective for cholinergic neurons in the MS-DBB produces a profound reduction of hippocampal theta during immobility or anesthesia, while relatively sparing theta associated with running and REM sleep.^41,42^ This non-cholinergic theta may depend upon septal GABAergic neurons because optogenetically silencing^43^ or selectively lesioning them^44^ eliminate REM- and locomotion-associated hippocampal theta. MS-DBB GABAergic neurons target hippocampal interneurons^45^ and are thought to pace the theta rhythm via periodic disinhibition of pyramidal cells.^46^ Besides the MS-DBB, lesioning the entorhinal cortex reduces theta throughout the hippocampus,^47^ and may contribute to theta rhythmicity.^48^ During active exploration, the place field-specific firing of CA1 pyramidal cells shows a dependence on theta phase, with neurons firing during earlier and earlier phases of theta as the rat moves through that cells’ place field,^49^ an effect that depends upon the integrity of the medial entorhinal cortex.^50^

Hippocampal theta oscillations appear to be traveling waves.^51, 52^ Recordings along the long axis of the hippocampus reveal a systematic progression of the phase of theta oscillations such that there is a 180 degree shift in theta phase from the septal to temporal pole, with the depolarizing phase leading in septal sites. Yet, local properties of theta such as peak frequency, coherence, and entrainment of unit activity are uniform throughout the hippocampus, which suggests that theta has similar effects on local processing. In particular, the depolarizing phase of the theta oscillation increases the strength of local gamma oscillations.^47^

The dorsal and ventral striatum exhibit theta activity during active waking states,^53–55^ but not REM sleep.^17,56^ These oscillations likely arise from hippocampal, not cortical, afferents.^55^ Both the striatal and hippocampal theta are associated with cyclical modulations of gamma power.^54^

With the exception of the prefrontal cortex, cortical sites generally do not show prominent theta oscillations.^57^ During performance of spatial tasks, prefrontal neurons fire in phase with hippocampal theta, but with a delay that implies a hippocampal to prefrontal directionality.^58^ This relationship is dependent on state; during REM sleep interactions between the prefrontal cortex and hippocampus are attenuated,^59^ similar to the striatum.^17^

**Fig. 3:**
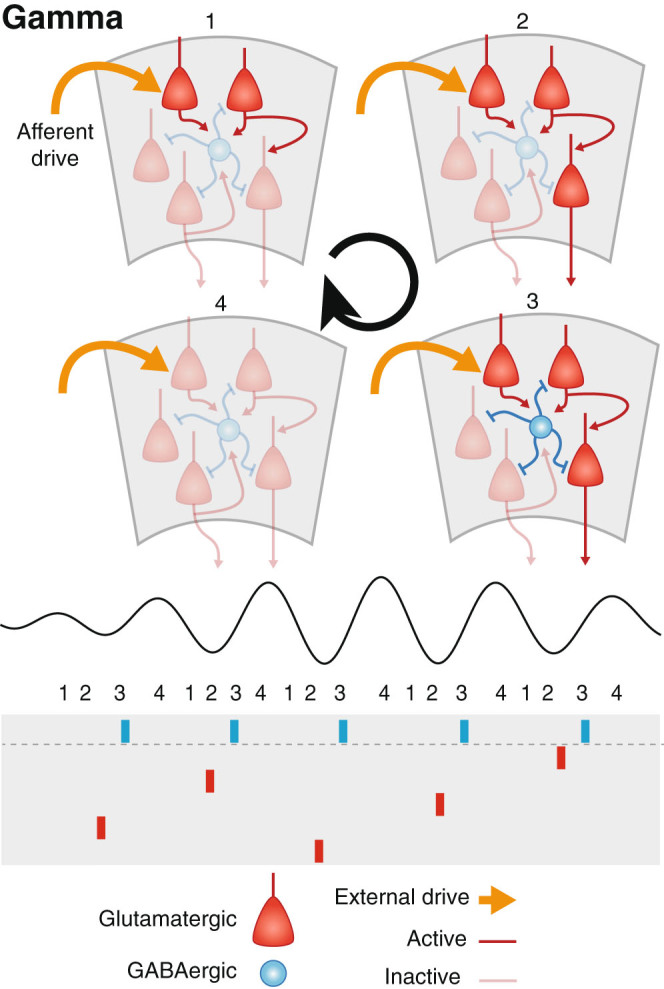
Gamma oscillations are generated in both hippocampus and cortex through the reciprocal interaction between excitatory pyramidal cells and inhibitory interneurons. *Top:* Excitation arising from extrinsic afferents and intrinsic connections (stages 1 and 2) activates local interneurons (3), which temporarily silences the local network (4). *Bottom:* Interneurons (*blue rasters*) tend to fire during every cycle of gamma, while pyramidal cells (*red rasters*) fire sparsely only every few cycles

Other cortical regions typically exhibit low amplitude high-frequency oscillations during waking states or REM sleep. This “activated” cortical state, which contains strong gamma band activity, can be induced by stimulation of the nucleus basalis magnocellularis,^60^ which is a posterior extension to the MS-DBB. Like hippocampal theta, the activated state is sensitive to cholinergic^60^ and GABAergic manipulations^61^ in nucleus basalis (Fig. 3).

## Generation of gamma oscillations

Gamma oscillations occur throughout the cortex, hippocampus, and striatum during waking and sleep. There are two dominant models for the genesis of gamma oscillations.^62^ One is based purely on interactions between inhibitory interneurons (Interneuron Network Gamma, ING). Such ING oscillations arise when a reciprocally connected population of inhibitory interneurons receives an exogenous excitatory drive, which does not need to be oscillatory. When this drive fires a sufficient number of interneurons simultaneously, they temporarily silence each other and come out of inhibition around the same time, priming them to fire synchronously again. Across several cycles of this, a substantial portion of the network becomes synchronized.^63^ ING is generally not considered to generate gamma oscillations in vivo, because in the intact brain interneurons are embedded in a network with pyramidal cells. The reciprocal interaction between these two subpopulations of neurons is responsible for the other mechanism of gamma generation (Pyramidal-ING, PING).

In PING oscillations, the excitatory drive is supplied by local pyramidal cells.^64^ Pyramidal cell spiking activates inhibitory interneurons, which temporarily silence the network by providing feedback inhibition to pyramidal cells and interneurons. As this inhibition wanes, pyramidal cells regain the ability to fire. Slight differences in excitatory drive onto particular subpopulations of pyramidal cells cause them to fire, which activates the diffuse inhibitory network and shuts down the rest of the network for another cycle. This process can effectively implement a winner-take-all calculation on each cycle.^65^

Both hippocampal and cortical microcircuits seem ideally suited to produce PING,^66^ as the connection probability between neighboring pyramidal cells and interneurons has been estimated at ~50–60%. Interneurons also exhibit dense connectivity, synapsing on each other and nearby pyramidal neurons, resulting in common oscillatory rhythms that entrain multiple functional subsets of pyramidal cells.^67^ Among the various interneuron subtypes, fast-spiking parvalbumin positive (PV) basket cells show the greatest entrainment by gamma.^68^ Optogenetically silencing them reduces the strength of gamma oscillations evoked by principal cell activation.^69^ Two forms of gamma, a slow (~35–60 Hz) and a fast (~60–100 Hz) type, have been reported in both cortical and hippocampal networks. In vitro, cortical slices can also exhibit distinct slow and fast gamma oscillations in supragranular and granular cortical layers, respectively. In addition the propensity of infragranular layers to display either rhythm is modulated by overall excitability.^70^ In the CA1 region in vivo, the occurrence of low- and high-frequency gamma oscillations is modulated by the phase of ongoing theta oscillations, and is associated with afferent drive arising from CA3 or the entorhinal cortex, respectively.^71^ While a thorough treatment of the functions of low and high gamma is beyond the scope of this review, it is worth noting that they have distinct behavioral correlates.^72,73^

The striatum also exhibits low and high gamma oscillations.^24, 74^ However, it remains unclear whether they are generated intrinsically or imposed by striatal afferents. Unlike cortex and hippocampus, there is only weak local connectivity between principal striatal neurons (MSNs), and these connections are GABAegic, reversing at −60 mV.^75^ Yet, the striatum also contains PV interneurons that synapse on one another and onto MSNs, in a manner similar to that seen in cortex.^76^ Thus, recurrent inhibitory connectivity among PV interneurons and MSNs could support ING oscillations. The other requirement for ING is afferent excitation, which could be provided by cortical or thalamic afferents.^77^ Alternatively, gamma oscillations could be imposed onto the striatum by cortical inputs, given that striatal PV interneurons fire in phase with cortical gamma oscillations.^74^

## Oscillations and memory systems

Three overarching principles emerge when we compare oscillatory activity across the memory systems considered in this review. First, gamma oscillations are present in all regions and coordinate local spiking, compressing it at the population level into short bursts. Second, gamma oscillations are modulated by the slower delta and theta oscillations. Third, oscillatory dynamics in these memory systems can operate in either a “slow” or “fast” mode. The slow mode happens during quiet rest or SWS and is characterized by large irregular activity in the hippocampus and delta oscillations in cortical and striatal circuits. The fast mode occurs during active waking and REM sleep and is typified by theta oscillations in the hippocampus and its targets, along with gamma oscillations in the rest of cortex.

Presumably, these state-dependent differences in oscillations across brain regions enable different mnemonic processes. The fast mode is associated with the efficacious encoding and retrieval of both declarative and procedural memories. On the other hand, during SWS the slow mode would regulate the offline consolidation of declarative memories, although some evidence indicates that during REM the fast mode promotes consolidation of procedural memories. Below, we consider evidence implicating oscillations in these different aspects of memory.

## Encoding and retrieval in the fast mode

Hippocampal theta has a well-established role in memory encoding.^78^ Elimination of theta by lesioning the MS-DBB disrupts the retention of a spatial foraging task.^79^ Moreover, tasks that tap into declarative memory are associated with enhanced theta coherence between the temporal lobe and hippocampal sites for items that were subsequently recalled.^80^ In patients undergoing presurgical mapping of epileptic foci with depth electrodes in the hippocampus, subsequent memory for image stimuli is associated with enhanced phase-locking between spiking and the local theta.^81^

Hippocampal theta is also associated with memory retrieval. Its coherence with the amygdala increases when rats exhibit freezing behavior associated either with aversive cues or contexts.^82^ The hippocampus conveys this theta signal to its targets. Indeed, theta coherence between the prefrontal cortex and hippocampus, as well as entrainment of prefrontal neurons by hippocampal theta, increases with learning, particularly at choice points in a memory-guided foraging task.^83,84^ In humans, theta oscillations occur throughout the temporal lobe and are strengthened following training on a virtual maze navigation task.^85^ Moreover, experimentally induced phase resetting of theta oscillations by entorhinal stimulation during the encoding of spatial landmarks enhances subsequent memory.^86^

The hippocampus projects to the ventral and medial portions of striatum,^87^ a connection that may supply spatial information. This pathway could link the hippocampal code for space with striatal coding of rewards and task conditions. During performance of a tone-cued T-maze task, as rats approached the decision point, medial striatum theta power and its coherence with hippocampus increases.^53,88^ Critically, mostly ventromedial striatal neurons are modulated by hippocampal theta.^55^ During reward periods, this modulation exhibits theta phase precession, highlighting the potential for phase-dependent computations between these systems.^89^

Theta oscillations often comodulate gamma as well, and many of the regions that exhibit learning-dependent modulation of theta show similar changes for gamma. With respect to retrieval, hippocampo-striatal gamma coherence increases during performance of a well-learned, cue-driven, operant response,^90^ and dorsal striatal gamma is modulated by the phase of hippocampal theta.^54^ In the human medial temporal lobe, hippocampo-entorhinal gamma coherence during encoding is stronger for subsequently recalled words,^80^ and gamma power is increased across the medial temporal lobe for recalled words.^91^

In contrast to the enhanced theta/gamma seen in medial temporal and frontal sites, occipital and parietal regions just show a boost in gamma^92,93^ for subsequently remembered items. Indeed, the earliest studies of learning-induced changes in the electroencephalogram (EEG) found that a loss of low-frequency components and emergence of low-voltage fast activity (so called “alpha-blocking”) accompanied learning and reflected increased attention.^94^ This activated state is thought to enhance the reliability of stimulus coding by sensory cortices,^95,96^ which may promote synaptic plasticity via spike timing-dependent mechanisms.^97,98^ In agreement with this, cue-induced gamma oscillations in the auditory cortex during fear conditioning predict both the resulting associative memory and plasticity.^99^ Regarding retrieval, like theta, cortical gamma oscillations are enhanced during behaviorally important stimuli,^100^ or incidental stimuli that are recognized from past experience.^101^

Enhanced gamma band activity was also shown to reflect successful encoding and retrieval in the hippocampus. In macaques, the strength of spike-field gamma coherence during the encoding of passively viewed novel pictorial stimuli predicted later recognition.^102^ With respect to retrieval, correct performance on an odor-sampling task in rodents was accompanied by increased coherence of pyramidal and interneuronal firing with theta and gamma field oscillations in CA1.^103^ These retrieval-related rhythms are likely acquired with training and may promote communication between the hippocampus and its downstream targets. In agreement with this notion, as rats acquire a novel odor–place association, they develop enhanced gamma coherence between the dorsal hippocampus and lateral entorhinal cortex during cue-sampling.^104^

In summary, both theta and gamma oscillations have the same relationships with encoding and retrieval across multiple forms of memory and brain regions. Moreover, these similarities exist despite regional differences in microcircuitry and information content.

## Consolidation with slow and fast modes

During SWS, the cortex exhibits large synchronous delta oscillations and a drastically reduced responsiveness to external stimuli.^105^ Yet, while this state appears to be the polar opposite of that seen during successful memory encoding, it plays an important role in memory consolidation. Cortical delta oscillations may be particularly important for this process, reflecting the reactivation and exchange of information between the cortex, striatum, and hippocampus.

Several lines of evidence point to the importance of delta oscillations for the consolidation of declarative memories. If a subject is presented with a set of stimuli to be subsequently recalled, an intervening sleep period will often increase the number of remembered items.^106^ Both the duration of SWS^107^ and the coherence of its delta oscillations^108^ positively correlate with memory retention. Importantly, manipulations, such as transcranial stimulation, that increase the strength of delta oscillations in cortex boost memory retention.^109^

In order for delta oscillations to enhance memory consolidation, they must somehow cause experience specific synaptic plasticity that affects behavior upon waking. This would likely depend on the reinstatement of cortical activity patterns associated with recent experience. Consistent with this notion, during SWS, cortical sites recently activated by sensory and motor experience exhibit enhanced delta power.^110–113^ In addition, following training on a visual discrimination task, delta waves tend to initiate in the occipital cortex and correlate with subsequent improvements in performance.^114^

Nested within these delta oscillations are upstates, which resemble cortical activation during waking, and could facilitate the transmission of information throughout cortical networks.^22^ In general, patterns of spontaneous ensemble activity during upstates match those observed during sensory coding.^115^ Critically, these ensemble activity patterns can be modified by experience. On a brain machine interface learning task, units selected for training are preferentially reactivated in SWS, presumably during upstates.^116^ Since cortical reactivations during upstates occur on a faster timescale than happen behaviorally,^117^ they could potentially engage synaptic plasticity mechanisms that depend upon the precise timing of pre- and postsynaptic spiking.

Delta oscillations and upstates throughout the striatum are also of potential importance to memory consolidation. In vitro, dorsal striatal upstates produce specific and repeatable sequences of ensemble activity similar to those seen in cortex.^118^ During SWS, ventral striatal neurons that were recently driven during task execution are reactivated, producing patterns of activity that reflect either the goal approach^119^ or reward delivery period.^56^

These reactivation events in cortex and striatum may in part be initiated by hippocampal SWRs. Consistent with this idea, SWRs occur during SWS and engender population activity that reflects previous experiences.^36^ Similarly, task-related medial prefrontal neurons, which entrain to hippocampal theta during task performance,^83^ exhibit significant activity modulation around SWRs during post-training sleep.^34^ Replay of recent experiences that coordinates with SWRs also occurs in visual cortex,^37^ and ventral striatum.^56^ However, in the latter case, replay events could also have been driven by coincident cortical upstates. Thus, delta oscillations during SWS may enhance memory consolidation by structuring the reactivation of experience-related neuronal ensembles in the cortex, striatum, and hippocampus.

SWRs also occur during quiet waking or pauses in behavior. Since these SWRs are associated with replay events in CA1 ensembles, they too may facilitate memory consolidation. Their occurrence during the consummatory phase of behavior,^38,39^ at which point reinforcement is often delivered, may strengthen memories by providing temporal contiguity between an immediately preceding experience and its present consequences. Indeed, disrupting these SWRs impairs the acquisition of a memory-guided spatial navigation task.^39^ While this consummatory replay tends to reflect the previous experience, anticipated circumstances are represented during awake SWR-associated replay immediately prior to initiation of task-related behaviors,^120^which could support memory retrieval, decision making, or consolidation.

In contrast to SWS, the role of REM and its associated theta oscillations in memory consolidation is less clear. While some studies have linked REM with the consolidation of procedural memories, others have not.^121^ Perhaps these discordant findings are explained by differences between the types of procedural memories under consideration. For instance, REM disruption does not affect consolidation of a skilled motor task,^122^ which depends partly on the dorsal striatum.^14^ This agrees with the fact that the striatum is not strongly coordinated with theta during REM sleep. Indeed no theta oscillations are seen in dorsal striatal MSNs recorded intracellularly during REM sleep,^17^ nor does the ventral striatum exhibit replay events during REM sleep following performance on a foraging task (in contrast to SWS).^56^ On the other hand, previous work has shown that the consolidation of emotional memory is susceptible to REM disruption.^43,123^ Perhaps not coincidentally, the amygdala, which is important for the consolidation of emotional memories,^124^ and receives direct ventral hippocampal and rhinal inputs,^125^ entrains to entorhinal theta during REM sleep^126^ and hippocampal theta under urethane anesthesia following the delivery of noxious stimuli.^127^ Moreover, during post-training REM sleep, the amygdala exhibits stronger theta interactions with the hippocampus in subjects that subsequently showed more robust fear memory.^128^ The same study did not find a similar enhancement between the hippocampus and medial prefrontal cortex. This agrees with a report showing that the medial prefrontal cortex is less sensitive to hippocampal inputs during REM theta.^59^ Recent human reports have found that sleep rich in REM does not enhance the consolidation of memories for the context surrounding an object, so-called source memory, which is thought to depend partly on the prefrontal cortex.^123^ Perhaps by cutting off cortex, subcortical structures monopolize the conversation with hippocampus.

What information could the hippocampus supply to subcortical regions, like the amygdala, during REM theta? The few data we have regarding this point suggest that CA1 broadcasts relatively veridical recreations of past experiences. As in SWS, CA1 exhibits replay events during REM, but unlike SWS these occur on a similar timescale as during the waking state.^129^ And while SWS replay is associated with CA3-driven SWRs, during REM, the influence of CA3 on CA1 appears weak, given the lack of SWRs and the low CA1–CA3 gamma coherence.^130^ Thus, it seems unlikely that the information stored in CA3 is relayed to CA1 during REM.

## Conclusion

Mnemonic processes are associated with different neuronal oscillations, but with remarkable consistency across different brain regions and types of memory. Theta and gamma oscillations prevail during the encoding and retrieval phases, and they are particularly pronounced during task-related stimuli. While boosts in gamma power are ubiquitous in the regions we considered, increases in theta are associated mainly with the hippocampus and its direct projection targets, such as the striatum and amygdala. During SWS, when memory consolidation processes take hold, delta oscillations seem to coordinate the exchange of information between the hippocampus and both the cortex and striatum, possibly supporting the consolidation of declarative and procedural memories. Although less is known about the physiology of memory consolidation during REM, the theta oscillations that prevail in that state might enable the hippocampus to transfer activity patterns related to past experiences to subcortical targets that mediate select forms of procedural memory, such as emotional memory.

Unfortunately, the vast majority of findings implicating brain oscillations in memory are correlational. Moving forward, it will be critical to test whether there is a causal link between oscillations and associated memory processes. A few recent studies have made significant progress on this front. For instance, Boyce *et al.* ^43^ showed that the consolidation of contextual memories is disrupted by interfering with hippocampal theta during REM, using close-loop optogenetic manipulations of the MS-DBB that did not disrupt REM sleep per se. Similarly, Miyamoto *et al.* ^131^ showed that the consolidation of perceptual memories during SWS could be dampened or enhanced by asynchronous or synchronous activation of related high- and low-order cortical areas at the delta frequency. These studies underscore the promise and potential of closed-loop optogenetic manipulations to assess the role of neuronal oscillations in memory. Future developments in real-time signal processing should add to the experimentalist’s toolkit,^132^ enabling control of spontaneously occurring oscillatory bursts. They should also allow for sculpting the phase and amplitude profiles of faster oscillations, such as gamma,^133^ which would open up new investigative possibilities to analyze the mechanisms of memory and neural computation in general.

## References

[1] Fries P (2015). Rhythms for cognition: communication through coherence. Neuron.

[2] Squire LR (1992). Declarative and nondeclarative memory: multiple brain systems supporting learning and memory. J. Cogn. Neurosci..

[3] Clark RE, Squire LR (1998). Classical conditioning and brain systems: the role of awareness. Science.

[4] Sherry DF, Schacter DL (1987). The evolution of multiple memory systems. Psych. Rev..

[5] Scoville WB, Milner B (1957). Loss of recent memory after bilateral hippocampal lesions. J. Neurol. Neurosurg. Psychiatry.

[6] Kim JJ, Fanselow MS (1992). Modality-specific retrograde amnesia of fear. Science.

[7] Alvarez P, Squire LR (1994). Memory consolidation and the medial temporal lobe: a simple network model. Proc. Natl Acad. Sci. USA.

[8] Sacco T, Sacchetti B (2010). Role of secondary sensory cortices in emotional memory storage and retrieval in rats. Science.

[9] Letzkus JJ (2011). A disinhibitory microcircuit for associative fear learning in the auditory cortex. Nature.

[10] Teixeira CM, Pomedli SR, Maei HR, Kee N, Frankland PW (2006). Involvement of the anterior cingulate cortex in the expression of remote spatial memory. J. Neurosci..

[11] Weinberger NM (2004). Specific long-term memory traces in primary auditory cortex. Nat. Rev. Neurosci..

[12] Trachtenberg JT (2002). Long-term in vivo imaging of experience-dependent synaptic plasticity in adult cortex. Nature.

[13] Sakai K, Miyashita Y (1991). Neural organization for the long-term memory of paired associates. Nature.

[14] Packard MG, Knowlton BJ (2002). Learning and memory functions of the basal ganglia. Annu. Rev. Neurosci..

[15] Steriade M, Nuñez A, Amzica F (1993). A novel slow (< 1 Hz) oscillation of neocortical neurons in vivo: depolarizing and hyperpolarizing components. J. Neurosci..

[16] Hayashi Y (2015). Cells of a common developmental origin regulate REM/non-REM sleep and wakefulness in mice. Science.

[17] Mahon S (2006). Distinct patterns of striatal medium spiny neuron activity during the natural sleep-wake cycle. J. Neurosci..

[18] Sheroziya M, Timofeev I (2014). Global intracellular slow-wave dynamics of the thalamocortical system. J. Neurosci..

[19] Sanchez-Vives MV, McCormick DA (2000). Cellular and network mechanisms of rhythmic recurrent activity in neocortex. Nat. Neurosci..

[20] Neske GT (2016). The slow oscillation in cortical and thalamic networks: mechanisms and functions. Front. Neural. Circuits.

[21] Haider B, Duque A, Hasenstaub AR, McCormick DA (2006). Neocortical network activity in vivo is generated through a dynamic balance of excitation and inhibition. J. Neurosci..

[22] Destexhe A, Hughes SW, Rudolph M, Crunelli V (2007). Are corticothalamic ‘up’ states fragments of wakefulness?. Trends Neurosci..

[23] Wilson CJ, Kawaguchi Y (1996). The origins of two-state spontaneous membrane potential fluctuations of neostriatal spiny neurons. J. Neurosci..

[24] Stern EA, Jaeger D, Wilson CJ (1998). Membrane potential synchrony of simultaneously recorded striatal spiny neurons in vivo. Nature..

[25] Mallet N, Le Moine C, Charpier S, Gonon F (2005). Feedforward inhibition of projection neurons by fast-spiking GABA interneurons in the rat striatum in vivo. J. Neurosci..

[26] Mahon S, Deniau JM, Charpier S (2001). Relationship between EEG potentials and intracellular activity of striatal and cortico-striatal neurons: an in vivo study under different anesthetics. Cereb. Cortex.

[27] Isomura Y (2006). Integration and segregation of activity in entorhinal-hippocampal subregions by neocortical slow oscillations. Neuron.

[28] Hahn TT, Sakmann B, Mehta MR (2006). Phase-locking of hippocampal interneurons’ membrane potential to neocortical up-down states. Nat. Neurosci..

[29] Hahn TT, Sakmann B, Mehta MR (2007). Differential responses of hippocampal subfields to cortical up-down states. Proc. Natl Acad. Sci. USA.

[30] Wolansky T, Clement EA, Peters SR, Palczak MA, Dickson CT (2006). Hippocampal slow oscillation: a novel EEG state and its coordination with ongoing neocortical activity. J. Neurosci..

[31] Vanderwolf CH (1969). Hippocampal electrical activity and voluntary movement in the rat. Electroencephalogr. Clin. Neurophysiol..

[32] Ylinen A (1995). Sharp wave-associated high-frequency oscillation (200 Hz) in the intact hippocampus: network and intracellular mechanisms. J. Neurosci..

[33] Battaglia FP, Sutherland GR, McNaughton BL (2004). Hippocampal sharp wave bursts coincide with neocortical “up-state” transitions. Learn. Mem..

[34] Peyrache A, Khamassi M, Benchenane K, Weiner SI, Battaglia FP (2009). Replay of rule-learning related neural patterns in the prefrontal cortex during sleep. Nat. Neurosci..

[35] Kudrimoti HS, Barnes CA, McNaughton BL (1999). Reactivation of hippocampal cell assemblies: effects of behavioral state, experience, and EEG dynamics. J. Neurosci..

[36] Girardeau G, Zugaro M (2011). Hippocampal ripples and memory consolidation. Curr. Opin. Neurobiol..

[37] Ji D, Wilson MA (2007). Coordinated memory replay in the visual cortex and hippocampus during sleep. Nat. Neurosci..

[38] Foster DJ, Wilson MA (2006). Reverse replay of behavioural sequences in hippocampal place cells during the awake state. Nature.

[39] Jadhav SP, Kemere C, German PW, Frank LM (2012). Awake hippocampal sharp-wave ripples support spatial memory. Science.

[40] Buzsáki G (2002). Theta oscillations in the hippocampus. Neuron.

[41] Lee MG, Chrobak JJ, Sik A, Wiley RG, Buzsáki G (1994). Hippocampal theta activity following selective lesion of the septal cholinergic system. Neuroscience.

[42] Kramis R, Vanderwolf CH, Bland BH (1975). Two types of hippocampal rhythmical slow activity in both the rabbit and the rat: relations to behavior and effects of atropine, diethyl ether, urethane, and pentobarbital. Exp. Neurol..

[43] Boyce R, Glasgow SD, Williams S, Adamantidis A (2016). Causal evidence for the role of REM sleep theta rhythm in contextual memory consolidation. Science.

[44] Yoder RM, Pang KC (2005). Involvement of GABAergic and cholinergic medial septal neurons in hippocampal theta rhythm. Hippocampus.

[45] Freund TF, Antal M (1988). GABA-containing neurons in the septum control inhibitory interneurons in the hippocampus. Nature.

[46] Robinson J (2016). Optogenetic activation of septal glutamatergic neurons drive hippocampal theta rhythms. J. Neurosci..

[47] Bragin A (1995). Gamma (40-100 Hz) oscillation in the hippocampus of the behaving rat. J. Neurosci..

[48] Quilichini P, Sirota A, Buzsáki G (2010). Intrinsic circuit organization and theta-gamma oscillation dynamics in the entorhinal cortex of the rat. J. Neurosci..

[49] O’Keefe J, Recce ML (1993). Phase relationship between hippocampal place units and the EEG theta rhythm. Hippocampus.

[50] Schlesiger MI (2015). The medial entorhinal cortex is necessary for temporal organization of hippocampal neuronal activity. Nat. Neurosci..

[51] Lubenov EV, Siapas AG (2009). Hippocampal theta oscillations are travelling waves. Nature.

[52] Patel J, Fujisawa S, Berényi A, Royer S, Buzsáki G (2012). Traveling theta waves along the entire septotemporal axis of the hippocampus. Neuron.

[53] DeCoteau WE (2007). Learning-related coordination of striatal and hippocampal theta rhythms during acquisition of a procedural maze task. Proc. Natl Acad. Sci. USA.

[54] Tort AB (2008). Dynamic cross-frequency couplings of local field potential oscillations in rat striatum and hippocampus during performance of a T-maze task. Proc. Natl Acad. Sci. USA.

[55] Berke JD, Okatan M, Skurski J, Eichenbaum HB (2004). Oscillatory entrainment of striatal neurons in freely moving rats. Neuron.

[56] Lansink CS (2008). Preferential reactivation of motivationally relevant information in the ventral striatum. J. Neurosci..

[57] Sirota A (2008). Entrainment of neocortical neurons and gamma oscillations by the hippocampal theta rhythm. Neuron.

[58] Siapas AG, Lubenov EV, Wilson MA (2005). Prefrontal phase locking to hippocampal theta oscillations. Neuron.

[59] Wierzynski CM, Lubenov EV, Gu M, Siapas AG (2009). State-dependent spike-timing relationships between hippocampal and prefrontal circuits during sleep. Neuron.

[60] Metherate R, Cox CL, Ashe JH (1992). Cellular bases of neocortical activation: modulation of neural oscillations by the nucleus basalis and endogenous acetylcholine. J. Neurosci..

[61] Kim T (2015). Cortically projecting basal forebrain parvalbumin neurons regulate cortical gamma band oscillations. Proc. Natl Acad. Sci. USA.

[62] Whittington MA, Traub RD, Kopell N, Ermentrout B, Buhl EH (2000). Inhibition-based rhythms: experimental and mathematical observations on network dynamics. Int. J. Psychophysiol..

[63] Wang XJ, Buzsáki G (1996). Gamma oscillation by synaptic inhibition in a hippocampal interneuronal network model. J. Neurosci..

[64] Börgers C, Kopell N (2003). Synchronization in networks of excitatory and inhibitory neurons with sparse, random connectivity. Neural. Comput..

[65] de Almeida L, Idiart M, Lisman JE (2009). A second function of gamma frequency oscillations: an E%-max winner-take-all mechanism selects which cells fire. J. Neurosci..

[66] Bartos M, Vida I, Jonas P (2007). Synaptic mechanisms of synchronized gamma oscillations in inhibitory interneuron networks. Nat. Rev. Neurosci..

[67] Perrenoud Q, Pennartz CM, Gentet LJ (2016). Membrane potential dynamics of spontaneous and visually evoked gamma activity in V1 of awake mice. PLoS Biol..

[68] Otte S, Hasenstaub A, Callaway EM (2010). Cell type-specific control of neuronal responsiveness by gamma-band oscillatory inhibition. J. Neurosci..

[69] Sohal VS, Zhang F, Yizhar O, Deisseroth K (2009). Parvalbumin neurons and gamma rhythms enhance cortical circuit performance. Nature.

[70] Ainsworth M (2011). Dual γ rhythm generators control interlaminar synchrony in auditory cortex. J. Neurosci..

[71] Colgin LL (2009). Frequency of gamma oscillations routes flow of information in the hippocampus. Nature.

[72] Tort AB, Komorowski RW, Manns JR, Kopell NJ, Eichenbaum H (2009). Theta-gamma coupling increases during the learning of item-context associations. Proc. Natl Acad. Sci. USA.

[73] Chen Z, Resnik E, McFarland JM, Sakmann B, Mehta MR (2011). Speed controls the amplitude and timing of the hippocampal gamma rhythm. PLoS ONE.

[74] Berke JD (2009). Fast oscillations in cortical-striatal networks switch frequency following rewarding events and stimulant drugs. Eur. J. Neurosci..

[75] Koós T, Tepper JM, Wilson CJ (2004). Comparison of IPSCs evoked by spiny and fast-spiking neurons in the neostriatum. J. Neurosci..

[76] Koós T, Tepper JM (1999). Inhibitory control of neostriatal projection neurons by GABAergic interneurons. Nat. Neurosci..

[77] Dubé L, Smith AD, Bolam JP (1988). Identification of synaptic terminals of thalamic or cortical origin in contact with distinct medium-size spiny neurons in the rat neostriatum. J. Comp. Neurol..

[78] Buzsáki G, Moser EI (2013). Memory, navigation and theta rhythm in the hippocampal-entorhinal system. Nat. Neurosci..

[79] Winson J (1978). Loss of hippocampal theta rhythm results in spatial memory deficit in the rat. Science.

[80] Fell J (2003). Rhinal-hippocampal theta coherence during declarative memory formation: interaction with gamma synchronization?. Eur. J. Neurosci..

[81] Rutishauser U, Ross IB, Mamelak AN, Schuman EM (2010). Human memory strength is predicted by theta-frequency phase-locking of single neurons. Nature.

[82] Seidenbecher T, Laxmi TR, Stork O, Pape HC (2003). Amygdalar and hippocampal theta rhythm synchronization during fear memory retrieval. Science.

[83] Benchanane K (2010). Coherent theta oscillations and reorganization of spike timing in the hippocampal-prefrontal network upon learning. Neuron.

[84] Jones MW, Wilson MA (2005). Theta rhythms coordinate hippocampal-prefrontal interactions in a spatial memory task. PLoS Biol..

[85] Kahana MJ, Sekuler R, Caplan JB, Kirschen M, Madsen JR (1999). Human theta oscillations exhibit task dependence during virtual maze navigation. Nature.

[86] Suthana N (2012). Memory enhancement and deep-brain stimulation of the entorhinal area. N. Engl. J. Med..

[87] Groenewegen HJ, Vermeulen-Van der Zee E, te Kortschot A, Witter MP (1987). Organization of the projections from the subiculum to the ventral striatum in the rat. A study using anterograde transport of Phaseolus vulgaris leucoagglutinin. Neuroscience.

[88] Thorn CA, Graybiel AM (2014). Differential entrainment and learning-related dynamics of spike and local field potential activity in the sensorimotor and associative striatum. J. Neurosci..

[89] van der Meer MA, Redish AD (2011). Theta phase precession in rat ventral striatum links place and reward information. J. Neurosci..

[90] Delcasso S (2014). Functional relationships between the hippocampus and dorsomedial striatum in learning a visual scene-based memory task in rats. J. Neurosci..

[91] Sederberg PB (2007). Hippocampal and neocortical gamma oscillations predict memory formation in humans. Cereb. Cortex.

[92] Sederberg PB, Kahana MJ, Howard MW, Donner EJ, Madsen JR (2003). Theta and gamma oscillations during encoding predict subsequent recall. J. Neurosci..

[93] Osipova D (2006). Theta and gamma oscillations predict encoding and retrieval of declarative memory. J. Neurosci..

[94] Thompson RF, Patterson MM, Teyler TJ (1972). The neurophysiology of learning. Annu. Rev. Psychol..

[95] Goard M, Dan Y (2009). Basal forebrain activation enhances cortical coding of natural scenes. Nat. Neurosci..

[96] Harris KD, Thiele A (2011). Cortical state and attention. Nat. Rev. Neurosci..

[97] Wespatat V, Tennigkeit F, Singer W (2004). Phase sensitivity of synaptic modifications in oscillating cells of rat visual cortex. J. Neurosci..

[98] Lee S, Sen K, Kopell N (2009). Cortical gamma rhythms modulate NMDAR-mediated spike timing dependent plasticity in a biophysical model. PLoS Comput. Biol..

[99] Headley DB, Weinberger NM (2011). Gamma-band activation predicts both associative memory and cortical plasticity. J. Neurosci..

[100] Headley DB, Weinberger NM (2013). Fear conditioning enhances γ oscillations and their entrainment of neurons representing the conditioned stimulus. J. Neurosci..

[101] Brunet NM (2014). Stimulus repetition modulates gamma-band synchronization in primate visual cortex. Proc. Natl Acad. Sci. USA.

[102] Jutras MJ, Fries P, Buffalo EA (2009). Gamma-band synchronization in the macaque hippocampus and memory formation. J. Neurosci..

[103] Rangel LM (2012). Rhythmic coordination of hippocampal neurons during associative memory processing. Elife.

[104] Igarashi KM, Lu L, Colgin LL, Moser MB, Moser EI (2014). Coordination of entorhinal-hippocampal ensemble activity during associative learning. Nature.

[105] Atienza M, Cantero JL, Escera C (2001). Auditory information processing during human sleep as revealed by event-related brain potentials. Clin. Neurophysiol..

[106] Fowler MJ, Sullivan MJ, Ekstrand BR (1973). Sleep and memory. Science.

[107] Diekelmann S, Biggel S, Rasch B, Born J (2012). Offline consolidation of memory varies with time in slow wave sleep and can be accelerated by cuing memory reactivations. Neurobiol. Learn. Mem..

[108] Mölle M, Marshall L, Gais S, Born J (2004). Learning increases human electroencephalographic coherence during subsequent slow sleep oscillations. Proc. Natl Acad. Sci. USA.

[109] Marshall L, Helgadóttir H, Mölle M, Born J (2006). Boosting slow oscillations during sleep potentiates memory. Nature..

[110] Vyazovskiy V, Borbély AA, Tobler I (2000). Unilateral vibrissae stimulation during waking induces interhemispheric EEG asymmetry during subsequent sleep in the rat. J. Sleep Res..

[111] Huber R, Ghilardi MF, Massimini M, Tononi G (2004). Local sleep and learning. Nature..

[112] Määttä S (2010). The effects of morning training on night sleep: a behavioral and EEG study. Brain Res. Bull..

[113] Wilhelm I (2014). Sleep slow-wave activity reveals developmental changes in experience-dependent plasticity. J. Neurosci..

[114] Mascetti L (2013). The impact of visual perceptual learning on sleep and local slow-wave initiation. J. Neurosci..

[115] Luczak A, Barthó P, Harris KD (2009). Spontaneous events outline the realm of possible sensory responses in neocortical populations. Neuron.

[116] Gulati T, Ramanathan DS, Wong CC, Ganguly K (2014). Reaction of emergent task-related ensembles during slow-wave sleep after neuroprosthetic learning. Nat. Neurosci..

[117] Euston DR, Tatsuno M, McNaughton BL (2007). Fast-forward playback of recent memory sequences in prefrontal cortex during sleep. Science.

[118] Carrillo-Reid L (2008). Encoding network states by striatal cell assemblies. J. Neurophysiol..

[119] Pennartz CM (2004). The ventral striatum in off-line processing: ensemble reactivation during sleep and modulation by hippocampal ripples. J. Neurosci..

[120] Diba K, Buzsáki G (2007). Forward and reverse hippocampal place-cell sequences during ripples. Nat. Neurosci..

[121] Walker MP, Strickgold R (2006). Sleep, memory, and plasticity. Annu. Rev. Psychol..

[122] Rasch B, Pommer J, Diekelmann S, Born J (2009). Pharmacological REM sleep suppression paradoxically improves rather than imapirs skill memory. Nat. Neurosci..

[123] Groch S, Zinke K, Wilhelm I, Born J (2015). Dissociating the contributions of slow-wave sleep and rapid eye movement sleep to emotional item and source memory. Neurobiol. Learn. Mem..

[124] McGaugh JL (2004). The amygdala modulates the consolidation of memories of emotionally arousing experiences. Annu. Rev. Neurosci..

[125] Pitkänen A, Pikkarainen M, Nurminen N, Ylinen A (2000). Reciprocal connections between the amygdala and the hippocampal formation, perirhinal cortex, and postrhinal cortex in rat. A review. Ann. N. Y. Acad. Sci..

[126] Paré D, Gaudreau H (1996). Projection cells and interneurons of the lateral and basolateral amygdala: distinct firing patterns and differential relation to theta and delta rhythms in conscious cats. J. Neurosci..

[127] Bienvenu TC, Busti D, Magill PJ, Ferraguti F, Capogna M (2012). Cell-type-specific recruitment of amygdala interneurons to hippocampal theta rhythm and noxious stimuli in vivo. Neuron.

[128] Popa D, Duvarci S, Popescu AT, Léna C, Paré D (2010). Coherent amygdalocortical theta promotes fear memory consolidation during paradoxical sleep. Proc. Natl Acad. Sci. USA.

[129] Louie K, Wilson MA (2001). Temporally structured replay of awake hippocampal ensemble activity during rapid eye movement sleep. Neuron.

[130] Montgomery SM, Sirota A, Buzsáki G (2008). Theta and gamma coordination of hippocampal networks during waking and rapid eye movement sleep. J. Neurosci..

[131] Miyamoto D (2016). Top-down cortical input during NREM sleep consolidates perceptual memory. Science.

[132] Grosenick L, Marshel JH, Deisseroth K (2015). Closed-loop and activity-guided optogenetic control. Neuron.

[133] Witt A (2013). Controlling the oscillation phase through precisely timed closed-loop optogenetic stimulation: a computational study. Front. Neural. Circuits.

